# 

**Published:** 1893-07

**Authors:** 


					﻿THE
Southern Medical¥ Record
A Monthly Journal of Medicine and Surgery.
i?OL. XXIII.	Atlanta, Ga., July, 1893.	NO 7?
EDITORS:
1. W. GRIGGS, M. D.	WILLIS F. WESTMORELAND, M. D.
[. mcfadden gaston, m. d. louis h. jones, m. d.
DAN. H. HOWELL, M. D., Bus. Mgr.
Entered at the Postoffice at Atlanta, Ga., as Second-Class Matter.
Mutual Printing Company, Publishers, 27 East Hunter St., Atlanta, Ga.
				

